# Severe Myalgic Encephalomyelitis/Chronic Fatigue Syndrome Leading to Assisted Suicide in a Patient in Her Late 30s: A Case Report

**DOI:** 10.7759/cureus.109798

**Published:** 2026-05-28

**Authors:** Dominika Opala, Erich Villiger, Ian Levenfus

**Affiliations:** 1 General Internal Medicine, MedVita Praxis, Obfelden, CHE; 2 General Medicine, MedVita Praxis, Obfelden, CHE; 3 General Medicine, Hausarztpraxis Altstetten, Zurich, CHE

**Keywords:** ebv positive, myalgic encephalomyelitis/chronic fatigue syndrome, physician-assisted suicide, postviral fatigue syndrome, severe fatigue

## Abstract

A patient in her late 30s developed severe myalgic encephalomyelitis/chronic fatigue syndrome (ME/CFS) following an Epstein-Barr virus infection. No distinct autoimmune or autoinflammatory disorder could be identified as the underlying cause of her symptoms, as the observed constellation of cytokine elevations (IL-2, IL-6, and IFN-γ) was not consistent with any known or established disease entity. Despite comprehensive multidisciplinary treatment over two years, including medical, psychological, and rehabilitative approaches, her condition deteriorated, and treatment-related hypersensitivities emerged. The severity and progressive nature of her symptoms, compounded by the absence of effective therapeutic options, ultimately led the patient to pursue assisted suicide.

## Introduction

Severe myalgic encephalomyelitis/chronic fatigue syndrome (ME/CFS) is a debilitating condition characterized by persistent and profound fatigue that is not alleviated by rest and is exacerbated by physical or mental activity, resulting in significant functional impairment. It is estimated that at least 60,000 individuals are affected by this condition in Switzerland alone [1], with around 25% being permanently bedridden. In approximately 60.3% of patients, a variety of infectious illnesses some time before the onset of ME/CFS was reported [2]. The most frequently reported was acute Epstein-Barr virus (EBV) infection, which occurred in 30% of infectious illnesses [2].

The precise pathophysiology remains unclear. Recently, the World Health Organization reviewed the literature on the classification of ME/CFS, including proposals to classify it as an infectious disease. As a result, the disease was retained within diseases of the nervous system [3]. Post-viral immune dysregulation, characterized by elevated cytokine levels and chronic low-grade inflammation, is considered a key factor. A meta-analysis of 42 studies from 2019 showed that patients with ME/CFS had significantly elevated tumor necrosis factor, IL-2, IL-4, transforming growth factor-β, and CRP levels [4]. In addition, the persistence of symptoms has been associated with autonomic nervous system dysfunction and abnormalities of the hypothalamic-pituitary-adrenal axis [5]. Several studies have reported a high prevalence of EBV viral capsid antigen (VCA) IgM antibodies in patients with ME/CFS, suggesting viral reactivation or a subclinical chronic infection. It has been proposed that EBV VCA IgM seropositivity may serve as a potential biomarker for identifying a distinct immunologically defined subset of patients with ME/CFS [6].

The primary objective of this case study is to emphasize the severity of progressively worsening symptoms associated with ME/CFS. Patients with this disease entity may experience a range of accompanying symptoms, including cognitive dysfunction, myalgia, arthralgia, and headaches. Cognitive impairment, often referred to as “brain fog,” is characterized by reduced concentration, memory deficits, and impaired executive function. According to the 2015 Institute of Medicine (IOM) criteria, ME/CFS is diagnosed based on the presence of three core symptoms persisting for more than six months: substantial activity reduction with profound fatigue, post-exertional malaise (PEM), and unrefreshing sleep, together with at least one additional symptom, either cognitive impairment or orthostatic intolerance. Symptoms must be present at least 50% of the time and be of moderate to severe intensity [7]. Severity of the disease, as defined by the International Consensus Criteria, includes mild (approximately 50% reduction in pre-illness activity level), moderate (mostly housebound), severe (mostly bedbound), and very severe (completely bedbound, requiring help with basic functions) categories [8].

In addition, this report highlights the paradoxical adverse outcomes of various treatments when not administered appropriately. The patient frequently felt misunderstood by healthcare professionals, who encouraged her to exceed her energy limits, which proved counterproductive. Finally, the patient was deemed to have decision-making capacity to determine whether to continue living or to end her life. Her decision-making capacity was assessed longitudinally during repeated clinical encounters and was considered preserved, as the patient consistently demonstrated the ability to understand, evaluate, and communicate informed healthcare decisions. The results of a US-based study suggest an increased risk of earlier suicide-related mortality among patients with ME/CFS. However, these findings should be interpreted with caution because of the small sample size and the potential overrepresentation of severely affected patients [9].

## Case presentation

A woman in her late 30s, with an academic background, presented with a variety of debilitating symptoms that developed following an acute EBV infection. Prior to the EBV infection, the patient was a socially and physically active individual. She had neither a significant past medical history of physical or mental illness nor a family history of neurological, psychiatric, or autoimmune disease, with no current or past use of prescription medications.

Upon presentation to the general practitioner’s office, the patient exhibited normal vital signs. Initial symptoms included severe fatigue, sore throat, dysphagia, and enlarged tonsils. Serological testing for IgM and IgG antibodies against EBV revealed a recent infectious mononucleosis infection. No focal neurological deficits, speech disturbances, memory impairment, confusion, psychosis, seizures, movement disorders, or psychiatric symptoms such as hallucinations or personality changes were observed. The level of consciousness remained normal.

Over a period of around two months, the patient developed different symptoms characteristic of ME/CFS. She complained of profound and persistent fatigue, dizziness, impaired concentration, increasing forgetfulness, intolerance to physical and mental exertion, as well as PEM. Furthermore, gastrointestinal symptoms such as nausea and constipation were reported. She reported experiencing disturbances in thermoregulation, characterized by a persistent sensation of coldness, particularly in her feet and hands. The patient’s fatigue did not subside with rest, and physical or mental activity exacerbated it. Moreover, she reported severe right-sided headaches, myalgia, and arthralgia, as well as sleep disturbances, including insomnia and nonrestorative sleep. Her skin became hypersensitive, particularly to water, and while showering was already uncomfortable, washing her hair proved to be especially painful.

At this stage, the patient fulfilled all diagnostic criteria for ME/CFS according to the 2015 Institute of Medicine/National Academy of Medicine criteria, which represent a commonly used and clinically applicable diagnostic framework for ME/CFS in general medical practice [10]. These criteria identify PEM as a core diagnostic feature, which was also present in the patient described. Two attempts to resume work, two and three months after the diagnosis of ME/CFS, resulted in a prolonged bedridden state that lasted for several weeks. The patient expressed frustration with her health status. She felt trapped in her body and could no longer participate in her favorite activities.

In order to exclude somatic causes of the chronic state of exhaustion, biochemical and immunoserological laboratory investigations were performed (Table 1, Table 2).

**Table 1:** Quantitative laboratory tests ordered throughout the course of the patient’s disease, before and after ME/CFS diagnosis Results outside the reference range are marked with an asterisk (*). ANAs, antinuclear antibodies; ATP, adenosine triphosphate; CMV, cytomegalovirus; EBV, Epstein-Barr virus; GM-CSF, granulocyte-macrophage colony-stimulating factor; INR, international normalized ratio; ME/CFS, myalgic encephalomyelitis/chronic fatigue syndrome; RF, rheumatoid factor; SAA, serum amyloid A; TNF-α, tumor necrosis factor alpha; VEGF, vascular endothelial growth factor

Laboratory test	Result	Unit	Reference range
EBV DNA-qPCR	0	IE/mL	<122
CMV IgG (ELISA)	<4	AE/mL	≤6
HIV Ag/Ab Combo quant	0.2	Quot.	<1.0
Anti-DFS70	3	U/mL	<20
Anti-double-stranded DNA	0	U/mL	<15
Anti-histone	0.4	Units	<1.0
Anti-chromatin	4	E/mL	<20
Anti-SS-A (Ro52 + Ro60)	0	E/mL	<10
Anti-SS-B (La, Ha)	0	E/mL	<10
ANAs	1:320*	Titer	<1:320
RF	<6	IU/mL	<10
MPO-ANCA (sensitive)	0	U/mL	<5
PR3-ANCA (sensitive)	0	U/mL	<3
Neopterin	1.22	ng/mL	<2.5
Classical pathway	66*	%	69-129
Alternative pathway	41	%	30-113
MBL pathway	45	%	10-125
IgA serum	3.13	g/L	0.7-4.0
IgM serum	1.4	g/L	0.4-2.8
IL-1β	<1.0	pg/mL	<1.2
sIL-2R in plasma	1017.4	U/mL	<1500
IL-2	15.15*	pg/mL	<8.89
IL-4	13.37*	pg/mL	<12.9
IL-6	10.5*	pg/mL	<8.0
IL-8	12.71	pg/mL	<13.87
IL-10	1.86	pg/mL	<5.76
IL-13	19.54*	pg/mL	<4.78
GM-CSF	10.72	pg/mL	<12.22
TNF-α	<2.81	pg/mL	<2.81
Gamma interferon (IFN-γ)	16.56*	pg/mL	<8.41
VEGF	38.34*	pg/mL	<8.43
RANTES	35.2*	ng/mL	<30.0
Anti-AT1R antibodies	6.2	U/mL	<10.0
Anti-ETAR antibodies	6.9	U/mL	<10.0
Anti-α1-adrenergic antibodies	3.2	U/mL	<7.0
Anti-β1-adrenergic antibodies	4.7	U/mL	<15.0
Anti-β2-adrenergic antibodies	4.1	U/mL	<8.0
Anti-muscarinic cholinergic receptor-2 antibodies	4.3	U/mL	<9.0
Anti-muscarinic cholinergic receptor-3 antibodies	2.8	U/mL	<6.0
Anti-muscarinic cholinergic receptor-4 antibodies	5	U/mL	<10.7
Anti-MAS1 antibodies	31.6*	U/mL	<25.0
Total histamine	81.2*	ng/mL	<65.5
Free spike protein	<4.5	pg/mL	<4.5
Intracellular ATP	3.14	µM	>2.5
CRP	<0.6	mg/L	<5
SAA	1.9	mg/L	<6.4
Ferritin	170	µg/L	13-150
Vitamin B12	414	ng/L	197-771
Folic acid	19	nmol/L	>8.8
INR	1.06	-	≤1.2
Prothrombin time	93	%	70-130
Leukotrienes C4, D4, and E4 in urine	847*	pg/mg creatinine	<385

**Table 2:** Qualitative laboratory tests ordered throughout the course of the patient’s disease, before and after ME/CFS diagnosis Results outside the reference range are marked with an asterisk (*). ANA, antinuclear antibody; ANCA, antineutrophil cytoplasmic antibodies; CMV, cytomegalovirus; EBNA, Epstein-Barr nuclear antigen; EBV, Epstein-Barr virus; ELISA, enzyme-linked immunosorbent assay; ME/CFS, myalgic encephalomyelitis/chronic fatigue syndrome; VCA, viral capsid antigen

Laboratory test	Result
EBV VCA IgG (ELISA)	Positive*
EBV VCA IgM (ELISA)	Positive*
EBV EBNA (ELISA)	Positive*
CMV IgM (ELISA)	Negative
HIV Ag/Ab combiscreen	Negative
SARS-CoV-2 nucleocapsid antibodies	Negative
ANA: cytoplasm	Negative
ANA pattern	Nuclear fine, speckled with weakly positive mitoses (non-AC pattern)
ANCA titer	Negative
IgG serum, including subclasses	In range
IL-5	Not detectable
Amyloid A	Negative
Complete blood count with differential	In range
Leukocyte subpopulations	In range
Urinalysis	In range
Metabolic and endocrine laboratory markers	In range

She was under outpatient psychiatric care and underwent detailed psychometric testing, which revealed 4 points on the Patient Health Questionnaire-9 and 10 points on the Beck Depression Inventory, indicating no evidence of depression. She scored 20 points on the David Bell scale and 58 points on the Fatigue Symptom Inventory, which demonstrated moderate to severe impairment of daily function due to fatigue, as well as 20 points on the Pittsburgh Sleep Quality Index, revealing poor sleep quality.

The blood count showed no evidence of anemia or any abnormal distribution of leukocyte subpopulations. Clinical chemistry revealed normal kidney and liver function, no evidence of iron deficiency or vitamin B12 or D deficiency, and normal thyroid function. The immunological laboratory diagnostics revealed only slightly elevated antinuclear antibodies (ANAs) and a reduced value of the classical pathway of complement activity. Further ANA differentiation revealed no evidence of systemic lupus erythematosus or Sjögren syndrome, since anti-double-stranded DNA, anti-histone, anti-chromatin, and anti-SSA/SSB were negative and were therefore considered nonspecific. A slightly reduced activity of the classical complement pathway is considered to have no pathological relevance in the absence of susceptibility to infections, especially those caused by encapsulated bacteria, and in the absence of evidence of complement consumption (C3c and C4), as well as given the potential for preanalytical variability.

The immunoglobulins (IgG, IgM, and IgA), including the IgG subclasses (IgG1-IgG4), were unremarkable. In conjunction with the generally unremarkable nature of the complement activity and the normal distribution of the lymphocyte subpopulations (T, B, and NK cells), an immune defect was considered highly unlikely. In cases where rheumatoid factor (RF) turned out to be negative, there was no evidence of diseases associated with RF, including rheumatoid arthritis. Negative antineutrophil cytoplasmic antibodies, in conjunction with the clinical presentation and the normal urine sediment, ruled out small-vessel vasculitis.

In order to search for a latent inflammatory state and an autoinflammatory syndrome, various inflammatory biomarkers were examined. Acute-phase proteins, including CRP, serum amyloid A, ferritin, and neopterin (typically associated with macrophage activation), were all within the normal range. The proinflammatory cytokines IL-1β, IL-5, and tumor necrosis factor ligand also measured within the reference range.

Sarcoidosis was considered unlikely because of normal neopterin and IL-2RL levels, no hypercalcemia, and no calcinuria. As states of exhaustion can be triggered by primary infections or reactivations of EBV or CMV, appropriate testing was carried out and revealed evidence of a past EBV infection without any active viral replication, as confirmed by PCR. In further clarification, small fiber neuropathy as well as multiple sclerosis were ruled out by unremarkable findings on cMRI and skin biopsy. The combination of the patient’s abnormal laboratory findings, including elevated IL-2, IL-6, interferon gamma, vascular endothelial growth factor, total histamine, CCL5 (RANTES), ANAs, and leukotrienes C4, D4, and E4, as well as reduced levels of complement components C3a and C4, does not correspond to any established rheumatologic or immunologic disease entity.

The management of the patient’s condition proved challenging because of the multifaceted nature of ME/CFS. A multidisciplinary approach was used, which included medical, psychological, and rehabilitative interventions (Table 3).

**Table 3:** Summary of complementary treatments administered throughout the patient’s disease course, with the appropriate indications

Therapy	Indication
Physiotherapy	Muscle pain and weakness
Psychotherapy	Depressive mood, chronic pain
Occupational therapy with pacing	Low energy limit
Cognitive behavioral therapy with a focus on activity pacing	Low energy limit
Acupuncture	Pain
Interval hypoxia-hyperoxia therapy	Low energy limit
Osteopathy	Pain
Buteyko breathing method	Low energy limit
Heparin-induced extracorporeal lipoprotein precipitation (plasma apheresis for microclots)	Low energy limit, gastrointestinal symptoms, thermoregulation disorder
Nutritional therapy	Malnutrition

The patient was prescribed a medication regimen designed to manage her symptoms, including analgesics, hypnotics, laxatives, antiemetics, and antidepressants (Table 4).

**Table 4:** Medications administered throughout the patient’s disease course

Medication name	Daily dose	Reason for prescription
Vitamin D3	20,000 IU	Malaise
Alprazolam	0.25 mg	Fear and panic attacks
Amitriptyline	25 mg	Pain
Rivaroxaban	10 mg	Thrombosis prophylaxis
Zolpidem	10 mg	Sleep disturbances
Cimetidine	200 mg	Gastrointestinal dysfunction
Paracetamol	500 mg on demand, maximum 3 g daily in total	Pain
Ibuprofen	400 mg on demand, maximum 1,200 mg daily in total	Pain
Pantoprazole	20 mg	Gastric protection during analgesia with NSAIDs
Trazodone	50 mg	Fear and panic attacks
Metamizole	500 mg on demand, maximum 3 g daily in total	Pain
Domperidone	10 mg on demand, maximum 30 mg daily in total	Nausea
Macrogol	13.125 g	Constipation
Mutaflor	-	Probiotic
High-calorie oral nutritional supplement	-	Malnutrition
Schüssler salts	-	Pain, immune support
Tramadol	50 mg on demand, maximum 100 mg daily in total	Pain
Omega-3 docosahexaenoic acid	-	Brain fog
Redormin	500 mg	Sleep disturbances
Mirtazapine	30 mg	Depressive mood

In addition to this, high doses of vitamin D were prescribed in an attempt to treat the patient’s general feeling of malaise, and antihistamine drugs were initiated to address the patient’s elevated histamine levels. Psychotropic drugs and sedatives were prescribed to treat sleep disturbances and panic attacks and to elevate the patient’s mood. At the patient’s request, heparin-induced extracorporeal low-density lipoprotein/fibrinogen precipitation (H.E.L.P.) plasma apheresis for microclots was also performed seven times, which the patient tolerated well. This led to short-term improvements in certain symptoms, including temperature perception and digestion, but did not alleviate exhaustion, headaches, or cognitive impairment.

Cognitive behavioral therapy with pacing and graded exercise therapy (GET) was recommended to facilitate a more comprehensive assessment of her energy balance and, by extension, enhance her overall functionality. However, the use of GET in severely affected patients with ME/CFS remains controversial due to possible symptom exacerbation in severely affected patients [11].

Accordingly, the 2021 National Institute for Health and Care Excellence (NICE) guideline no longer recommends GET as a curative treatment for ME/CFS and instead emphasizes individualized energy management strategies [11]. Acupuncture was applied for pain management. Interval hypoxia-hyperoxia therapy (IHHT) and the Buteyko breathing technique were recommended to support energy metabolism and reduce malaise. Nutritional support was also provided to treat her malnutrition and promote weight gain. Several of these approaches, including IHHT and H.E.L.P. apheresis, remain empirical or experimental, with limited supporting evidence. Overall, these interventions demonstrated either no effect or only short-term symptom improvement.

**Figure 1:**
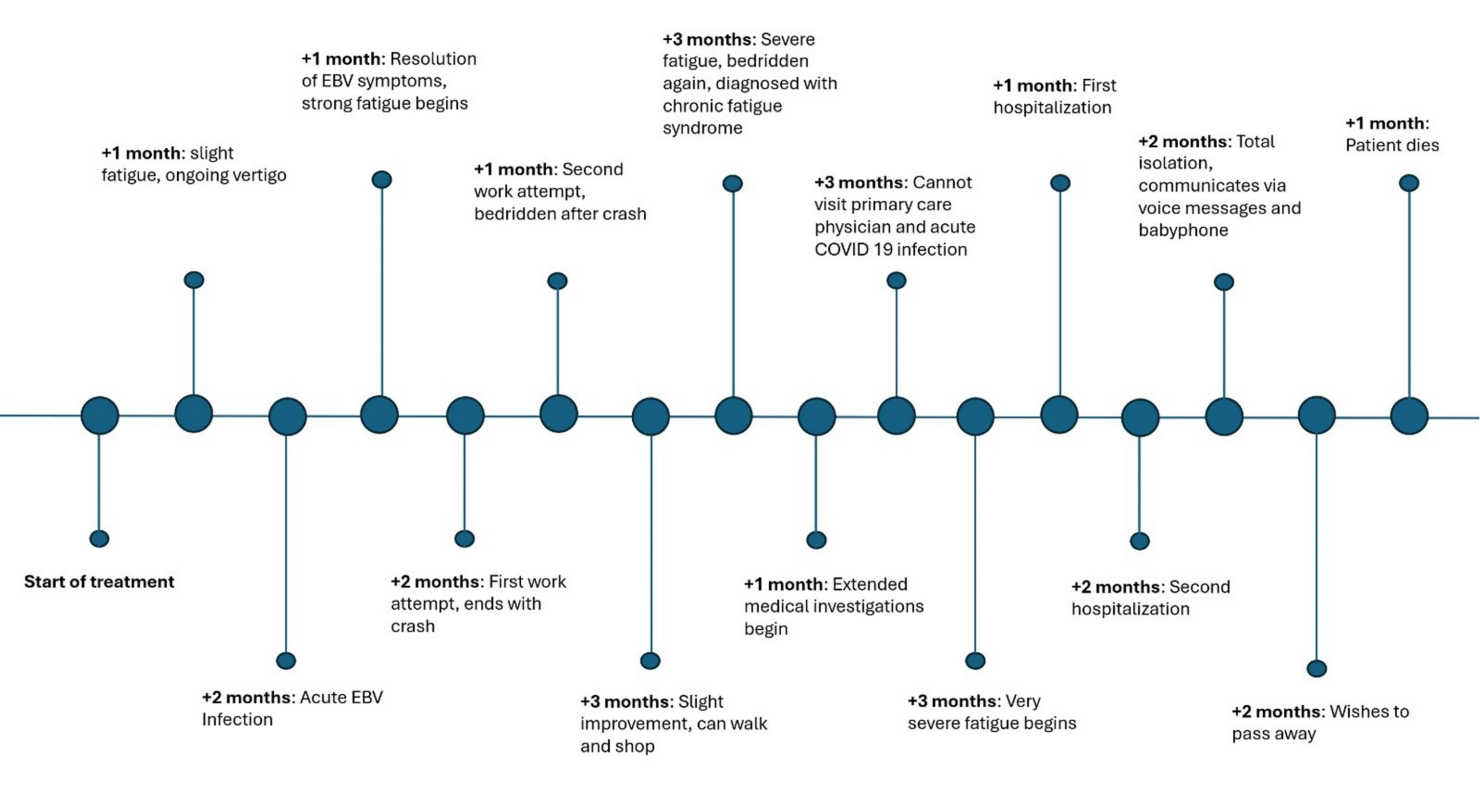
Medical history timeline focusing on the patient’s energy level and severity of the disease EBV, Epstein-Barr virus The figure was created using Microsoft PowerPoint (Microsoft Corporation, Redmond, WA, USA).

The patient’s condition progressively deteriorated, as shown in Figure 1.

During the initial three months after acute EBV infection, the patient was able to concentrate for a duration of about 25 minutes, perform simple household activities, and walk a few kilometers. Attempts to resume work resulted in “crashes,” characterized by profound physical and cognitive fatigue occurring when an individual’s energy limit is exceeded. During this phase, the patient remained in a recumbent position for extended periods ranging from days to weeks, unable to tolerate light, noise, physical exertion, or even the presence of visitors. Following each crash, the patient’s energy levels never fully rebounded to their previous state.

After a six-month period from the acute EBV infection, the patient encountered significant challenges in performing basic household tasks or engaging in any form of employment. Headaches and back pain reached intolerable levels, despite the augmentation of analgesic therapy. The patient also reported dizziness, excessive sweating, orthostatic intolerance, heightened sensitivity to noise and light, as well as a thermoregulatory disorder. Her social life was significantly affected. At the time, she took analgesics and antiemetics as required.

A trial of physiotherapy was undertaken, but her energy limits were frequently exceeded, resulting in repeated crashes and increased muscle pain. Furthermore, the patient commenced psychological support, attending weekly sessions; however, she often encountered a lack of understanding from healthcare personnel at all levels.

One year later, her daily capacity for reading and listening to music was no more than 10 minutes, requiring frequent breaks. At this stage, even a 15-minute consultation with the family doctor was a considerable challenge for the patient, both cognitively and physically. At this stage, a formal diagnosis of ME/CFS was established. She consulted an immunologist, a rheumatologist, a neurologist, a neuroimmunologist, and a specialist in ME/CFS. Despite ongoing care, her condition worsened, and three months later, she was confined to her home. At this point in her disease course, the patient experienced her first infection with SARS-CoV-2, which triggered markedly severe fatigue.

She maintained telephone consultations with her psychologist, which lasted approximately seven minutes, the maximum she could tolerate. Furthermore, she was no longer able to take advantage of the specific consultation hours with other medical specialists and therapists. The patient needed help with all daily activities, including personal hygiene. She could barely eat, needing antiemetics before each meal. Her cognitive function was compromised, with a maximum capacity to concentrate for seven minutes, five times a day. She slept irregularly and exhibited severe sensitivity to light and sound. In the final two months of her life, she did not tolerate direct human contact, not even with her parents. The patient began experiencing panic attacks with increasing frequency.

Over the course of the illness, the patient was hospitalized on two occasions in an acute internal medicine ward, primarily due to increasing malnutrition, nausea, and chronic diffuse pain. Her body mass index decreased over a period of 18 months from 18 to 14.2 kg/m².

Psychologically, the patient experienced considerable distress due to her chronic illness and cognitive impairment, often referred to as “brain fog.” She reported feelings of frustration and hopelessness, especially since her condition had not improved despite numerous medical interventions. The patient’s expressions of hopelessness were specifically related to the irreversible progression of debilitating symptoms, loss of autonomy, and anticipated decline, without evidence of global negative self-appraisal or diminished interest in non-illness-related domains. The affective responses were congruent with the clinical situation and fluctuated in accordance with symptom burden and physical functioning, rather than showing the persistence, pervasiveness, or affective flattening typical of a depressive disorder. The number and severity of her symptoms and the perception that available interventions were ineffective [12,13] led the patient to the decision to pursue assisted suicide, which was eventually accepted by her family members, primary care physician, and a panel of medical assessors from the Swiss assisted suicide association (EXIT).

The patient’s psychological status and cognitive function were thoroughly evaluated, and she was deemed competent by independent physicians to make her own decisions about her life.

## Discussion

The diagnosis of ME/CFS is primarily clinical and is based on a patient’s medical history and symptoms. Physical examination should rule out signs of endocrine, malignant, cardiopulmonary, and neuromuscular disorders. Reactivation of a preexisting EBV infection or a subclinical chronic course may act as a potential trigger for the onset of ME/CFS [6]. In the present case, however, it remains uncertain whether the patient had a prior EBV infection before the onset of symptoms, as no baseline serological data are available.

The prevalence of ME/CFS, estimated in December 2023, is 1.3% of adults, with women (1.7%) being almost twice as frequently affected as men (0.9%) [14]. ME/CFS should not be confused with burnout or other psychiatric disorders [1]. The precise etiology remains unclear, and some patients, with their atypical presentations, are not adequately recognized and stigmatized in conventional medicine [15]. Not feeling understood by others, as well as being told that the disease is of psychological origin, may also have a substantial impact on suicidal thoughts, as other studies have shown [16]. The available treatment options are limited, do not facilitate recovery, and generally focus on symptom relief [15]. ME/CFS places a significant burden on healthcare systems due to the need for extensive examinations and prolonged treatment [12]. Statistics reveal that spontaneous recovery in ME/CFS patients is observed in only approximately 10% of cases [17]. US studies show that all-cause mortality in chronically exhausted patients is not higher than expected, but the suicide rate is significantly higher than in the general US population [12]. A very severe disease course associated with a comparable somatic and psychological burden in the absence of prior vaccination has been described in the literature [18,19].

## Conclusions

The primary objective of this case report is to emphasize the severity of illness and the complex medication and treatment history, which regrettably resulted in the patient’s premature death through assisted suicide after a two-year disease course. Throughout the course of her illness, the patient underwent repeated psychometric evaluations that consistently revealed no evidence of clinically significant depression. She remained under continuous psychological and general medical care until the end of her life and repeatedly expressed that, given her profound loss of quality of life, she no longer wished to continue living. Throughout this process, she demonstrated preserved decision-making capacity and understanding of the consequences of her decisions. This case does not suggest assisted suicide as an expected outcome of ME/CFS but rather illustrates the potential consequences of severe disease combined with therapeutic limitations and psychosocial burden.

The clinical presentation and disease course, marked by the rapid deterioration of the patient’s energy limits, underscore the challenges in the management of ME/CFS. Careful pacing and avoidance of interventions that may exceed the patient’s energy limits and trigger clinical worsening are crucial in treatment management. The profound fatigue can lead to social isolation and limit the ability to engage in medical appointments and further investigations. Moreover, the heightened sensitivity to various stimuli, including therapeutic intervention, can result in paradoxical adverse reactions, such as the aforementioned crashes. The report also highlights the importance of early diagnosis in preventing disease progression and guiding appropriate treatment strategies, as emphasized in the NICE guidelines. Finally, this case illustrates the urgent need for greater awareness, improved diagnostic pathways, compassionate patient-centered care, and the development of evidence-based therapies for severe ME/CFS.
